# Equal access, (Un)equal uptake: a longitudinal study of cataract surgery uptake in older people in England

**DOI:** 10.1186/1472-6963-14-447

**Published:** 2014-09-30

**Authors:** Jennifer Whillans, James Nazroo

**Affiliations:** The Cathie Marsh Institute for Social Research (CMIst), School of Social Sciences, University of Manchester, Humanities Bridgeford Street, Manchester, M13 9PL UK

**Keywords:** Wealth, Social position, National Health Service (NHS), Visual impairment

## Abstract

**Background:**

Uptake of cataract removal is a function of the effectiveness of the healthcare delivery services: services that are inaccessible, inappropriate, or unaffordable will not be utilised by (sub)populations, who consequently live with untreated cataracts. The aim of the study was to identify the relationship between individual wealth inequalities and uptake of cataract surgery in England, having controlled for the effects of potentially confounding variables.

**Methods:**

The final sample comprised of 2091 respondents from the English Longitudinal Study on Ageing (ELSA) who were diagnosed with cataracts prior to or during the study, aged 50 and over at wave 1, who had not undergone cataract surgery prior to the first survey observation, and had also provided a response in the second wave of the study. The uptake of cataract surgery was measured using the question, *have you ever had cataract surgery?* Data from waves 1-5 were used to identify those having received treatment during the 8-year observation window of ELSA. Survival analysis techniques were used.

**Results:**

Having controlled for the effects of potentially confounding variables, wealth did not make a statistically significant contribution to the overall fit of the Cox proportional hazard model nor were individual parameters statistically significant. Thus, respondents’ socioeconomic position was not found to be a significant predictor in the uptake of cataract surgery in the UK. Receiving a recommendation from a medical professional was a key driving factors in the uptake of cataract surgery.

**Conclusions:**

Study findings suggest that uptake of cataract surgery among over 50s with a cataracts diagnosis in England do not discriminate on the grounds of individuals’ material social position (wealth).

## Background

Cataracts remain the leading cause of visual impairment among older people worldwide [1]. Surgical removal of the cataract with intraocular lens implantation remains the only effective treatment available to restore or maintain vision [1–4]. In England, cataract surgery is the most common elective surgical procedure performed under the publically-funded National Health Service (NHS). The uptake of treatment by those who need or are eligible for surgery is argued to be a function of the effectiveness of the healthcare delivery services: services that are inaccessible, inappropriate, or unaffordable will not be utilised by (sub)populations, who consequently live with untreated cataracts [5].

The picture of inequities in utilisation of medical care is not clear [6, 7]. Some research suggests that health care utilisation in the general population in the UK is relatively equitable [7, 8], while a number of other macro-studies of utilization (measuring the use of all NHS services) and micro-studies (studying the use of particular services or treatments) indicate inequality, with access to specialist services on the NHS being pro-rich [7, 9, 10]. Reconciling findings may also be challenging as some studies approach socio-economic inequalities in access to care and treatment using individual-level characteristics (in terms of income, education, and economic activity) while others use geographic variables (index of multiple deprivation (IMD)). Where cataract surgery uptake is the specific focus of enquiry, Kennan et al. have argued that the rate of cataract surgery by local authority showed a positive correlation with the index of multiple deprivation (IMD) such that the greater the deprivation in an area, the higher the rate of cataract surgery [11]; this suggests that, under the NHS, access to care seems not to be significantly compromised in socially deprived local authorities. This finding does not take into account other socioeconomic factors that may be influencing these observed gradients [12], and is based on aggregate, geographical data rather than individual markers of need. Using longitudinal data and survival analysis techniques, this study aims to identify whether individual wealth inequalities lead to an unequal uptake of cataract surgery among older people in England, having controlled for the effects of other socioeconomic and medically relevant factors.

## Methods

The English Longitudinal Study of Ageing (ELSA) contains detailed information on the health, economic, and social circumstances of older people in England [13] in order that the effects of social, behavioural, and economic factors upon age-related changes in the health and wellbeing of older people can be assessed. The core sample was initially drawn from households that had previously responded to the Health Survey for England (HSE) in 1998, 1999, or 2001 and that were aged 50 and over at the beginning of the data collection period. Five waves of data have been collected to date, beginning in 2002, and collected at 2-year intervals. ELSA contains information on the uptake of cataract surgery irrespective of whether it was carried out under publically-funded or private care, providing a complete picture of access to cataract surgery. This study involved the analysis of a secondary data source. At the time of data collection ethical approval for all the ELSA waves was granted from the National Research and Ethics Committee. Informed consent was gained from all participants.

### Assessment of dependent variable

In each wave, ELSA respondents were asked, *have you ever had cataract surgery?* In Wave 1, this variable was used to identify individuals entering the studying having already undergone cataract treatment. The data from subsequent waves was used to identify those having received treatment during the 8-year observation window of ELSA.

### Exclusionary criteria

First, a set of exclusionary criteria were used to ensure that the analysis took into account the probability of cataract surgery uptake given that a respondent was eligible for surgery (i.e. they had cataracts), in order that uptake rates can be disentangled from differential prevalence rates [12, 14]. Of the initial sample of 11,391 core respondents, 3159 respondents either reported having cataracts on entry to the study (N = 1543) or reported a new diagnosis in waves 2 to 5 (N = 1616). Second, respondents were excluded if they entered the study having already had cataract surgery, i.e. the event being examined had already occurred (N = 784). It was also necessary for respondents to be observed at wave 2, which excluded a further 284 respondents. The final sample comprised of 2091 respondents who were diagnosed with cataracts prior to or during the study, had not undergone cataracts surgery prior to the first survey observation, and had also provided a response in the second wave of the study.

### Assessment of economic circumstances

Wealth at baseline was used as an objective measure of respondents’ economic circumstances. Income was not suitable as only a small proportion of people over the age of 65 are in employment and income predictably decreases substantially once individuals retire and leave the labour market [15–17]. On the other hand, wealth is argued to reflect older peoples’ life-time cumulative social status and indicates command over material resources better than any other measure of socioeconomic status in later life [18, 19]. Wealth is measured in net total non-pension wealth at benefit unit (household) level, which includes the value of the primary house minus the outstanding primary house mortgage, the value of savings and shares minus credit card debts and loans, and the value of other properties and businesses. Wealth was entered into the model as quintiles with the highest wealth quintile as the reference group.

### Assessment of other covariates

Increasing age is not only associated with the presence of cataract, but also with the severity of visual impairment from cataract. To control for the non-linear effect of age, age groups were entered into the model. Respondents were grouped into 10-year bands according to age at wave 1 (Age 50-59, 60-69, 70-79, and 80 and over); for interpretation is should be noted that those in the cohort ’50-59 years at wave 1’ were aged 58-67 in wave 5. The youngest cohort was used as the reference category. Gender was not anticipated to be a significant factor in the uptake of cataract surgery in England, unlike in middle and low-income countries [20], but is nevertheless included in the analysis for the sake of completeness. Ethnicity (white, other) was also entered in the models.

While in England there is a universal free health service on which cataract surgery is available, over 12% of the population is covered by voluntary health insurance schemes, known in the UK as private medical insurance (PMI) [21]. The decision to buy PMI is linked with income [22] and, in the US, type and level of insurance has been shown to be associated with differential cataract surgery uptake rates [23–26]. Although potentially related to wealth, the effects of PMI on uptake of cataract surgery were controlled for by entering a binary variable into the models (no private insurance, private insurance).

To control for the effects of geographical variation and neighbourhood deprivation on access to care [11], the Index of Multiple Deprivation (IMD) was used, which is a measure based on distinct dimensions of deprivation that can be measured separately at the small-area level. Seven dimensions of deprivation are included: income deprivation; employment deprivation; health deprivation and disability; education, skills, and training deprivation; barriers to housing and services; living environment deprivation; and crime. IMD was entered into the models as quintiles with the least deprived quintile as the reference group.

The effects of respondent’s self-reported baseline (preoperative) visual function were controlled for (Excellent, very good, good, fair, poor or blind) [27] and furthermore the effects of a recommendation for surgery from a medical professional (no recommendation, recommended, recommendation not known). Respondents were only questioned about recommendation for cataract surgery in waves 2 and 5; there were a notable proportion of respondents with a response of ‘item not applicable’ or with missing data, which together have been categorised as ‘recommendation not known’.

### Data analysis

Survival analysis techniques were performed using Stata, version 12.1. All analyses were conducted using wave 2 weights adjusting for survey non-response (all respondents had wave 2 weights but did not necessarily participate beyond this point). When using the *stset* command in Stata to indicate the format of the data, the *enter(time exp)* option was used to define when a subject came under observation for the uptake of cataract surgery; i.e. when they had received a cataract diagnosis and were therefore at risk of, or eligible for, treatment. Respondents with a diagnosis prior to the start of the survey (N = 565) were eligible for surgery throughout the 8-year study period while respondents receiving a new diagnosis during the study (N = 1526) would only be eligible from the point of diagnosis.

First, life tables were calculated using Kaplan-Meier estimates to describe the distribution of cataract surgery uptake over time. All respondents were considered eligible for surgery from the point of diagnosis of cataracts until the occurrence of surgery was reported, a censoring event, or the final wave of the study. Kaplan-Meier failure function curves were examined to make univariate comparisons of discrete groups of respondents for all of the categorical predictors. Cox regression-based tests were then performed as a statistical evaluation for the equality of survival curves and as an indicator of the suitability of each variable for inclusion in subsequent models (rather than using logrank tests as data were weighted); predictors were considered for inclusion if the test had a p-value of 0.25 or less. This univariate analysis was supplemented by basic descriptive statistics to examine the distribution of the outcome variable among all respondents.

Second, Cox proportional hazards models were used to analyse the effect of wealth on the uptake of cataract surgery, while controlling for the effects of a number of other potentially significant risk factors. Starting with a null model, predictors were entered incrementally into the model; nested models were compared using likelihood ratio tests to assess to overall contribution of the newly entered set of variables.

## Results

During the 8-year follow up period, of the 2091 respondents, 740 reported a diagnosis of cataracts and subsequently underwent cataract surgery (34.9%); 1407 respondents (65.5%) were diagnosed with cataracts prior to or during the study but did not undergo cataract surgery, with 902 not receiving treatment during the study, and 449 leaving the study without first having reported treatment (Tables 1 and 2). Table 2 shows the flow of respondents into and out of the analysis. In the interval between waves 1 and 2, 1015 were eligible for surgery and 220 received treatment; there was a net loss of -215 respondents as 167 respondents did not proceed in the study beyond this point and 382 respondents were added following a newly reported cataract diagnosis. The data for the periods between waves 2 and 3, 3 and 4 and 4 and 5 can be read in a similar way. The overall probability of undergoing cataract removal was 0.557 (Table 2).

**Table 1:** **Sample characteristics and incidence of surgery**

	Baseline/totals		Underwent cataract surgery	
	N	Weighted %	N	Weighted %
Gender				
Male	751	35.8	280	36.9
Female	1,340	64.2	460	35.3
Age group				
50-59	307	14.8	72	23.6
60-69	689	30.8	227	33.5
70-79	771	37.5	302	39.7
80 and over	324	16.8	139	42.3
Ethnicity				
Non-white	50	3.1	21	39.9
White	2,041	96.9	719	35.7
Wealth quintile				
Highest	441	19.8	168	38.5
Fourth	390	18.0	122	31.0
Middle	424	20.3	154	37.2
Second	407	19.7	133	32.1
Lowest	409	21.2	160	40.7
Missing	20	1.0	3	16.2
Private health insurance				
No	1,881	90.3	650	35.0
Yes	210	9.7	90	43.5
Index of multiple deprivation				
Least deprived	461	21.2	171	37.1
Second	521	24.0	176	34.8
Middle	439	21.1	152	34.6
Fourth	372	18.5	128	35.7
Most deprived	298	15.2	113	37.8
Self-reported vision				
Excellent	209	9.7	69	32.8
Very good	546	25.5	166	30.9
Good	872	41.3	293	34.1
Fair	344	17.1	151	43.8
Poor or blind	120	6.4	61	50.6
Treatment recommended				
No recommendation	386	18.30	49	13.1
Recommended	368	18.10	237	64.4
Not known	1,337	63.60	454	34.3

**Table 2:** **Life table of cataract surgery uptake**

Time	Interval	Beg. total	Uptake	Net lost	Uptake (failure) Function weighted	Uptake (failure) Function unweighted	[95%	Conf. Int.]
1	[1, 2)	1015	220	-215	0.230	0.217	0.193	0.243
2	[2, 3)	1010	165	-178	0.358	0.345	0.318	0.373
3	[3, 4)	1023	182	-234	0.471	0.461	0.434	0.489
4	[4, 5)	1075	173	902	0.557	0.548	0.522	0.574

**Figure 1:**
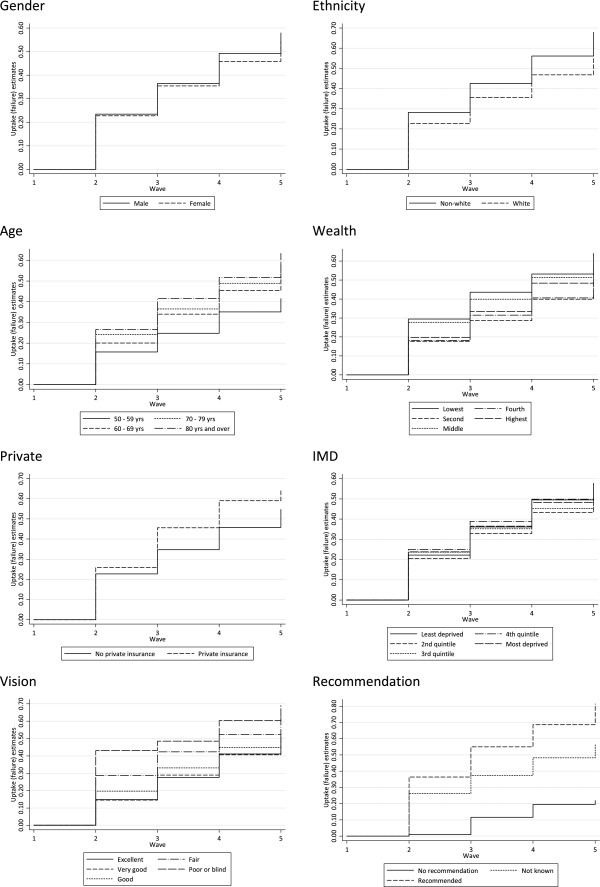
**Kaplan-Meier failure function curves.**

Performing basic descriptive statistics (Table 1) and plotting Kaplan–Meier uptake (failure) function by each of the categorical predictors (Figure 1) suggest that there is no discernible relationship between respondents’ level of wealth and treatment uptake; the lowest wealth quintile were the most likely to undergo cataract removal (40.7%), followed by the middle, highest, second, then fourth quintile. On the other hand, age, ethnicity, private insurance, preoperative vision, and receiving a recommendation for treatment appeared to be related to the uptake of treatment. As age at baseline increased, so the probability of undergoing cataract surgery increased. Ethnicity appeared to have an effect of treatment uptake with non-white respondents being more likely to undergo surgery compared with white respondents. Private medical insurance appeared to influence the likeliness of cataract surgery with 35.0% of those without insurance having cataract surgery and 43.5% with private insurance undergoing surgery. As baseline visual impairment increases so the probability of undergoing cataract surgery increases. Finally, receiving a recommendation from a medical professional for cataract removal increases the probability of surgery uptake whereas having cataracts, but having no recommendation for a health profession reduces the likeliness of proceeding with cataract removal (64.4% and 13.1% respectively).

Multivariate Cox proportional hazard models were used to estimate independent associations between wealth and the uptake of cataract surgery, controlling for the effects of potentially confounding variables. Cox regression-based tests for equality of survival curves indicated that all variables were suitable for inclusion in Cox proportional hazards models with the exception of IMD, which was unlikely to contribute anything to the final mode (p = .715), and was therefore not included (Table 3). Model 0 was the null model. Model 1 included wealth alone. Demographic variables were then entered into model 2 (gender, ethnicity, age), then other economic factors into model 3 (private insurance) and, finally, medically-relevant factors into model 4 (self-reported vision and professional recommendation). Likelihood ratio tests comparing nested models showed that each set of newly entered variables made a significant contribution to the overall explanatory power of the model. Wealth was dropped from model 5 and likelihood ratio tests comparing model 5 nested within model 4 indicated that having controlled for the effects of all other variables, wealth was not a significant contribution to the overall fit of the model (LR chi^2^ = 10.54, p = 0.061).

**Table 3:** **Cox regression-based tests for equality of survival curves**

	Wald Chi-Square	P-value
Gender	1.59	0.208
Age group	19.84	0.000
Ethnicity	1.57	0.211
Wealth	18.36	0.003
Private insurance	5.70	0.017
IMD	2.12	0.715
Self-reported vision	22.83	0.000
Recommendation	181.22	0.000

Likelihood ratio tests comparing nested Cox proportional hazard models indicated that the inclusion of wealth in the model increased the explanatory power of the model, compared to the null model (LR chi2 = 164.11, p < 0.000); however, when entered as the final variable in the model it did not offer a significant contribution (LR chi^2^ = 10.54, p = 0.061). The coefficients for wealth did not reveal an informative pattern and none were statistically significant (in either model 1 or model 4). Furthermore, gender and ethnicity were not found to be a significant factor in the uptake of cataract surgery, having controlled for the effects of other variables (Table 4).

**Table 4:** **Cox proportional hazard model**

	HR	95% CI	P
Gender			
Male	1	.	.
Female	0.879	(0.768 - 1.007)	0.063
Ethnicity			
Non-white	1	.	.
White	0.941	(0.587 - 1.510)	0.802
Age group			
50-59	1	.	.
60-69	1.476	(1.155 - 1.885)	0.002
70-79	1.599	(1.257 - 2.034)	0.000
80 and over	1.750	(1.343 - 2.281)	0.000
Wealth			
Highest	1	.	.
Fourth	0.868	(0.696 - 1.081)	0.206
Middle	1.053	(0.866 - 1.281)	0.602
Second	0.860	(0.698 - 1.059)	0.156
Lowest	1.132	(0.922 - 1.389)	0.236
Missing	0.482	(0.160 - 1.450)	0.194
Private			
No private insurance	1	.	.
Private insurance	1.213	(0.991 - 1.484)	0.061
Self-reported vision			
Excellent	1	.	.
Very good	0.920	(0.710 - 1.192)	0.529
Good	1.007	(0.787 - 1.289)	0.957
Fair	1.229	(0.932 - 1.621)	0.143
Poor	1.457	(1.041 - 2.040)	0.028
Treatment recommended			
No recommendation	1	.	.
Recommended	5.490	(4.110 - 7.333)	0.000
Not known	3.533	(2.639 - 4.730)	0.000

On the other hand, age was a significant factor in the uptake of cataract surgery and with increasing age. A gradient in hazard ratio was seen in the effect of self-reported vision at baseline (preoperative) on the uptake of cataract surgery; however, only the effect of self-reporting poor vision of blindness at baseline had a significant effect on the uptake of treatment (HR = 1.457, p = 0.028). Receiving a recommendation for cataract surgery by a medical professional significantly increased the likeliness of undergoing treatment (HR = 5.490, p < 0.000).

## Discussion

The study findings suggest that there is no clear relationship between respondents’ wealth and the uptake of cataract surgery among over 50s with a cataract diagnosis in England. Descriptive statistics showed that the lowest wealth quintile were the most likely to undergo cataract surgery, but Cox proportional hazards models indicated that this was not statistically significant having controlled for the effects of other variables. Increasing age and having self-reported poor preoperative vision were related to undergoing treatment but receiving a recommendation for treatment from a medical professional was a particularly strong influencing factor in the uptake of surgery (HR = 5.490).

The pathway to cataract surgery under the NHS begins with a diagnosis and subsequent referral for surgery initiated either by an optometrist or general practitioner (GP), offering a clear explanation of the magnitude and significance of this variable. However, there is a possible wealth effect on, first, receiving a diagnosis (and therefore for inclusion in the study sample) and, second, on the receipt of a recommendation for surgery [9, 28]. Older people in lower income brackets are the least likely to attend NHS-funded eye examinations where identification of eye disease and diagnosis of cataracts are made (citing the potential subsequent cost of glasses as a barrier) [29]. Once in attendance of health services, Dixon et al [7] suggest that by virtue of their education, articulacy, and general self-confidence, the better off may be better at persuading healthcare professionals that their needs can only be met through specialist services and intervention. While the present study does not reveal significant wealth inequalities in uptake of cataract surgery among those eligible, wealth inequalities may exist further upstream in the process [6, 7]. Thus while recipients of NHS cataract treatment do not face fee-for-service charges, individual-level socioeconomic factors may still (indirectly) shape treatment uptake.

The use of private health care by those who can afford it may distort utilisation rates where data are available for NHS care only [6]; the absence of data from the private sector in Keenan et al. may partly explain the socioeconomic gradient observed [11] and has also been raised as a major limitation in the study by Judge et al [10] who examine equity in access to joint replacement in England. ELSA is not restricted to surveying publically-funded care but also includes surgery conducted in the private sector, making this a distinct strength of this study. Private Medical Insurance (PMI) was entered as a variable in the models and while it made a significant contribution to the overall fit of the model, the test of the individual parameter itself was not significant (at p < .05). By comparison, in the United States where there is a patchwork of public and private health insurance across the population, inequality in access to care leads to differential cataract surgical uptake [24–26]. This may be because publically funded Medicare insurance to cover cataract surgery still entails a financial burden on the individual in the form of deductibles, co-payments, and charges not covered; as such, a beneficiary’s income was associated with the odds of undergoing cataract surgery, suggesting an unequal access to care due to financial barriers [23]. A key concern not addressed by this paper is the timing of treatment and the time elapsed between cataract diagnosis and surgery. Notwithstanding the reduction in waiting times for cataract surgery on the NHS between 1997 and 2007 [30], it is highly likely that while these factors are not associated with uptake of treatment (i.e. whether or not surgery occurs), patients in some Primary Care Trusts and those with PMI (and the financial means to pay for this cover) will likely have shorter waiting times. ELSA does not contain information on the date of diagnosis nor the date of treatment; with 2-year intervals between waves of data collection, an analysis of waiting times using ELSA is rather imprecise, although an analysis of this kind would be a valuable next step. In conclusion, these study findings reassuringly suggest that in England, the absence of a fee-for-service charge on the NHS appears to remove financial barriers to the uptake of treatment and recommendation for treatment (which will necessarily be related to preoperative vision) is the driving factor; however, wealth effects may exist in diagnosis of cataracts (a precursor to treatment) and in waiting times leading to treatment, by virtue of differences experienced under public and private care.

## Conclusion

The study findings suggest that uptake of cataract surgery among over 50s with a cataract diagnosis in England do not discriminate on the grounds of individuals’ material social position (wealth). Further research is required into whether there is a wealth effect on, first, receiving a diagnosis and, second, on the receipt of a recommendation for surgery which may indirectly lead to wealth inequalities.
